# Construction and Validation of a Convenient Clinical Nomogram to Predict the Risk of Brain Metastasis in Renal Cell Carcinoma Patients

**DOI:** 10.1155/2020/9501760

**Published:** 2020-11-17

**Authors:** Yuexin Tong, Zhangheng Huang, Chuan Hu, Changxing Chi, Meng Lv, Youxin Song

**Affiliations:** ^1^Affiliated Hospital of Chengde Medical University, Chengde, Hebei 067000, China; ^2^Qingdao University Medical College, Qingdao, Shandong 266000, China; ^3^Yunnan Cancer hospital, Kunming, Yunnan 650000, China

## Abstract

Brain metastasis (BM) is a typical type of metastasis in renal cell carcinoma (RCC) patients. The early detection of BM is likely a crucial step for RCC patients to receive appropriate treatment and prolong their overall survival. The aim of this study was to identify the independent predictors of BM and construct a nomogram to predict the risk of BM. Demographic and clinicopathological data were obtained from the Surveillance, Epidemiology, and End Results (SEER) database for RCC patients between 2010 and 2015. Univariate and multivariate logistic regression analyses were performed to identify the independent risk factors, and then, a visual nomogram was constructed. Multiple parameters were used to evaluate the discrimination and clinical value. We finally included 42577 RCC patients. Multivariate logistic regression analysis showed that histological type, tumor size, bone metastatic status, and lung metastatic status were independent BM-associated risk factors for RCC. We developed a nomogram to predict the risk of BM in patients with RCC, which showed favorable calibration with a *C*-index of 0.924 (0.903-0.945) in the training cohort and 0.911 (0.871-0.952) in the validation cohort. The calibration curves and decision curve analysis (DCA) also demonstrated the reliability and accuracy of the clinical prediction model. The nomogram was shown to be a practical, precise, and personalized clinical tool for identifying the RCC patients with a high risk of BM, which not only will contribute to the more reasonable allocation of medical resources but will also enable a further improvements in the prognosis and quality of life of RCC patients.

## 1. Introduction

Renal cell carcinoma (RCC) is a common malignancy of the genitourinary system [1], with over 400,000 new cases and 17,000 RCC-associated mortalities in 2018 worldwide [2]. Approximately one-quarter of patients with RCC have metastatic disease at the time of diagnosis, and another 35% of them will develop distant metastases (DMs) during the process of tumor progression [3, 4]. Brain metastasis (BM) is a typical type of metastasis in RCC patients. In a study conducted by Bianchi et al., it was shown that the rate of BM ranged from 2% to 16% in metastatic RCC (mRCC) [5]. Although noticeable progress has been made in tumor treatment during the last several decades, renal cell carcinoma with brain metastasis (RCCBM) exhibits a limited response to current anticancer treatment methods [6–8]. BM is still thought to be closely related to mortality for patients with advanced-stage RCC [9]. The median overall survival of RCC patients with BM has been described as only 5-8 months [10, 11]. Thus, RCCBM is considered a significant issue in RCC studies.

The evaluation of BM in RCC may help improve clinical outcome and perhaps contribute to decreasing the potential risks of aggressive multimodality treatment required for advanced-stage cancer. Verma and his colleagues found that the use of tyrosine kinase inhibitors (TKIs) influenced the natural disease course and prognosis of RCC by preventing the development of BM [12]. Thus, the improved understanding and surveillance of BM will be beneficial for improving long-term prognosis for RCC patients. Some exploratory-stage predictors have been reported to evaluate the risks of BM in RCC. A retrospective study on 52 RCCBM patients revealed that smoking cigarettes and lung metastases were highly associated with the RCCBM [13]. However, no studies have focused on the development of an ideal predictive model for predicting the risk of BM in RCC, which means that the probability of BM cannot be quantified. In other words, the performance of BM-related factors in the prospective prediction of BM in RCC patients is still unknown. Due to the rarity of BM in RCC, obtaining adequate cases from our clinical practice to conduct the current study was extremely difficult. Thus, we used the Surveillance, Epidemiology, and End Results (SEER) database, a commonly used tool to study rare tumors, which provides data from 18 cancer registries and includes approximately 30% of the United States population. Therefore, the purpose of this study was to establish and validate a clinical prediction model to quantifying the risk of BM for RCC patients based on the SEER database. This study will help to promote personalized treatment and medical decision-making for patients with RCC.

## 2. Materials and Methods

### 2.1. Study Population Selection

**Figure 1:**
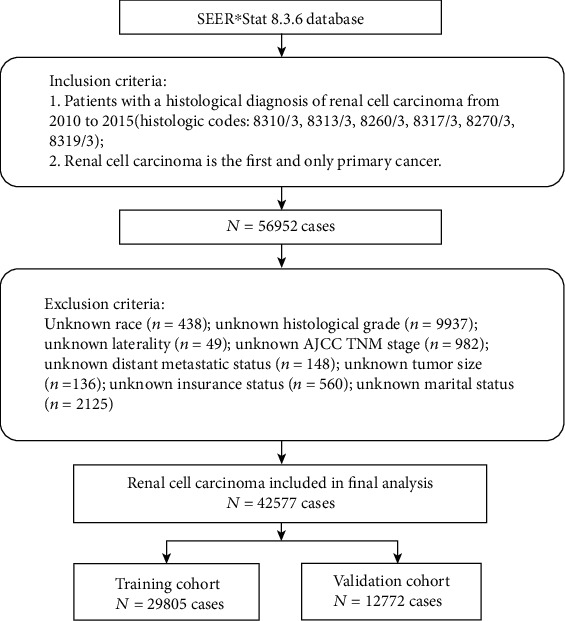
The diagram of the process of patient selection.

The study population was derived from the SEER database, and the data were downloaded with SEER∗Stat software version 8.3.6. The SEER program is an open access database which sponsored by the National Cancer Institute that contains demographic and clinicopathological information on cancer incidence and survival from 18 population-based cancer registries. The inclusion criteria were as follows: (1) patients with a histological diagnosis of RCC from 2010 to 2015 (histologic codes: 8310/3, 8313/3, 8260/3, 8317/3, 8270/3, and 8319/3); (2) RCC was the first and only primary cancer; patients with important clinical information on age, race, sex, histological type, histological grade, laterality, T stage, N stage, tumor size, insurance status, marital status, and distant metastatic status (brain, lung, bone, and liver) were unknown and excluded from this study. Finally, a total of 42577 RCC patients were identified to evaluate risk factors for the development of BM in patients with RCC and construct a diagnostic nomogram. In addition, this study was exempt from a medical ethics review and did not require informed consent because the data extracted from the SEER database were anonymized and deidentified prior to release.  Figure 1 shows the process of patients' selection procedure in this study.

### 2.2. Data Elements

Based on accessible demographic data and tumor clinicopathological data recorded in the SEER database and previous studies, we extracted 14 variables from the SEER database that could be potentially associated with the development of BM in RCC patients. Demographic variables included age, sex, race, insurance status, and marital status. Cancer characteristics included the tumor size, histological grade, histological type, laterality, T stage, and N stage. In addition, we included data on metastases, including liver metastases (yes or no), lung metastases (yes or no), and bone metastases (yes or no). Histological type were defined by ICD-O-3 codes: clear cell (8310/3, 8313/3), papillary (8260/3), chromophobe (8317/3, 8270/3), and collecting duct (8319/3). All patients in this study were staged using the American Joint Committee on Cancer TNM staging system, 7th edition.

### 2.3. Statistical Analysis

R software (version 3.6.1) and SPSS 25.0 were used for all statistical analyses in this study. All patients were divided into training (*n* = 29805) and validation (*n* = 12772) cohorts with a ratio of 7 : 3. The classification process was completely randomized and performed with R software. The diagnostic nomogram was constructed based on patients in the training cohort and tested by patients in the validation cohort. Univariate logistic analysis was performed to identify BM-associated risk factors. Variables with a *P* value < 0.05 in univariate analysis were further incorporated into multivariate logistic regression analysis to identify the independent risk factors for BM in RCC patients. A multivariate logistic regression model was developed to quantify the relationship between BM and the potential characteristics that were meaningful in univariate analysis. The diagnostic nomogram was constructed with the “rms” package in R software based on the independent risk factors for BM in HCC patients. Meanwhile, a receiver operating characteristic (ROC) curve was plotted, and the area under the curve (AUC) was used to show the discrimination of the diagnostic nomogram. The AUCs of each independent risk factor were also compared with the AUC of the predictive nomogram. Moreover, a calibration curve was generated and decision curve analyses (DCA) were performed to evaluate the diagnostic nomogram. In the present study, a *P* value < 0.05 (two-sided) was considered statically significant.

## 3. Results

### 3.1. Baseline Characteristics

Between 2010 and 2015, a total of 42577 patients diagnosed with RCC were included in this study cohort, of whom 228 patients had BM. In the training cohort, the majority were White people in race distribution (82.53%), and 19039 patients (63.88%) were male. Most of RCC patients (97.35%) received external economic support. As regards tumor characteristics, the most common T stage and N stage were T1-2 (79.60%) and N0 (97.17%), respectively. There was no significant difference in laterality, with left accounting for 49.38% and right accounting for 50.62% of RCC patients. The proportion of patients with clear cell RCC in this study was 79.01%. The detailed baseline characteristics for all patients are shown in Table 1.

### 3.2. Risk Factors for BM in RCC Patients

To identify the BM-associated variables in RCC patients, 14 variables were analyzed. Different variables were classified according to whether the patient had BM or not. In the training cohort, univariate analysis identified race (*P* = 0.009), histological grade (*P* < 0.001), T stage (*P* < 0.001), histological type (*P* < 0.001), N stage (*P* < 0.001), bone metastasis (*P* < 0.001), liver metastasis (*P* < 0.001), lung metastasis (*P* < 0.001), and tumor size (*P* < 0.001). Subsequently, the above variables were further included in multivariate logistic regression analysis, which showed that histological type (*P* = 0.005), bone metastasis (*P* < 0.001), lung metastasis (*P* < 0.001), and tumor size (*P* < 0.001) were independent predictors for BM in RCC patients. Details are presented in Table 2.

### 3.3. Development and Validation of a Diagnostic Nomogram for BM in RCC Patients

**Figure 2:**
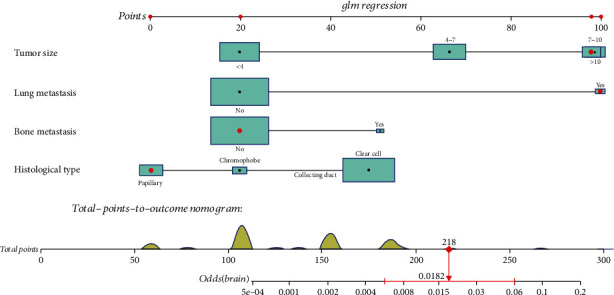
A nomogram prediction model for risk of BM in patients with RCC.

**Figure 3:**
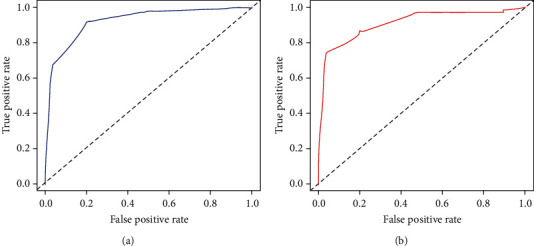
Receiver operating characteristic (ROC) curves and area under curve (AUC) of the nomogram for predicting BM in patients with RCC in the training cohort (a) and the validation cohort (b). The AUC was used to show the discrimination of the nomogram.

**Figure 4:**
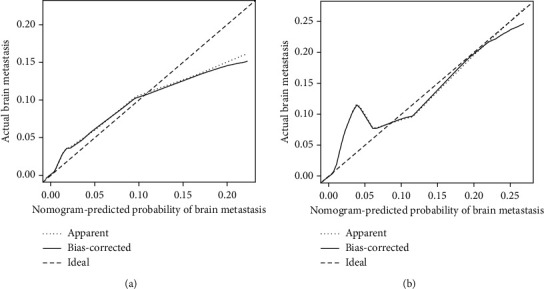
Calibration curves of the nomogram for predicting BM in patients with RCC in the training cohort (a) and the validation cohort (b). The *x*-axis represents the nomogram-predicted probability of BM; the *y*-axis represents the actual probability of BM. Plots along the 45-degree line indicate a perfect calibration model in which the predicted probabilities are identical to the actual outcomes.

**Figure 5:**
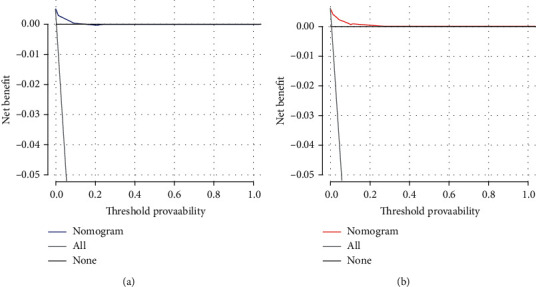
Decision curve analysis (DCA) of the nomogram for predicting BM in patients with RCC in the training cohort (a) and the validation cohort (b). This diagnostic nomogram shows a notable positive net benefit, indicating that it has a good clinical utility in predicting estimating the risk of BM in patients with RCC.

**Figure 6:**
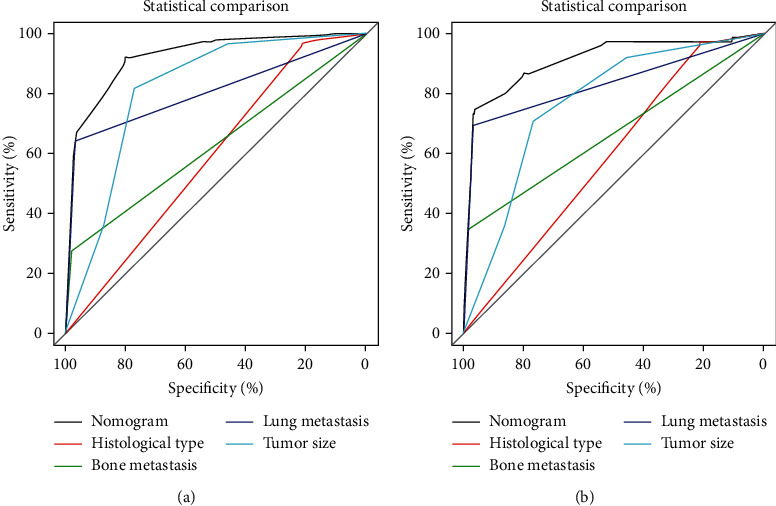
Comparison of AUC between the predictive nomogram and each independent predictor in the training cohort (a) and the validation cohort (b).

Based on multivariate logistic regression analysis, a diagnostic nomogram was constructed to predict the risk of BM in RCC patients (Figure 2). In the diagnostic nomogram, values for individual patients are located along the variable axes, and a line is drawn upward to the point axis to determine the number of points assigned for each variable. There is a total point line at the bottom of the nomogram, and each variable score is summed to give the total points. Then, a vertical line is drawn from the total point scale to the BM axis to obtain the probability. To assess the accuracy and validity of the model, ROC curves are plotted in Figure 3, with AUC in the training and validation cohorts of 0.924 (0.903-0.945) and 0.911 (0.871-0.952), respectively, indicating that the risk model has a better discriminative ability. The calibration curve showed high consistency between the observed and predicted results (Figure 4). In addition, the DCA results suggested that the nomogram was a good diagnostic tool for the risk of developing BM in patients with RCC (Figure 5). Moreover, as shown in Figure 6, ROC curves of each independent BM-associated risk factor were also generated in this study. The results showed that the AUC of the comprehensive nomogram was higher than the AUC of any single independent predictor.

## 4. Discussion

In recent decades, considerable advances in tumor therapy have significantly improved the overall survival of mRCC patients [14, 15]. BM in RCC is still an important topic in the field of kidney malignancy research. Although the occurrence of BM is relatively low, it was reported that the presence of BM signified a worse prognosis compared with the lung or bone metastases [16]. A recent retrospective study suggested that the median overall survival of RCCBM patients was only 8.2 months [17]. However, the early detection of BM is likely a crucial step for RCC patients to receive appropriate treatment and prolong their overall survival. Nevertheless, brain imaging is not routinely recommended for all RCC patients based on surveillance guidelines from the American Urologic Association, European Association of Urology, and National Comprehensive Cancer Network, unless clinical or laboratory evidence indicates a high risk of BM for individual patients [18–20].

It therefore seems significant for clinical decision-making and personalized management to explore BM-related predictors in RCC patients and identify RCC patients with a high risk of BM. A conventional study reported that tumor size and age were risk factors for BM in RCC [21]. However, to date, a convenient predictive model for predicting BM in RCC has not been developed, which means that the personal risk of BM cannot be accurately estimated by combining all independent risk factors for BM. Nomograms are an easy-to-understand and multivariate visualization model to predict and quantify the incidence of a specific clinical outcome for individual patients and are widely applied in various malignancies [22–24]. Each independent risk factor included in the model was given a weighted point value to represent its effect on BM in RCC. This tool could be used to provide a reference for scientific and rational clinical decisions and promote the development of precision cancer medicine.

In our study, the analysis based on the SEER database from 2010 to 2015 indicated that histological type, tumor size, bone metastatic status, and lung metastatic status were independent risk factors for BM in RCC. There might be a relationship between histological grade, race, T stage, N stage, liver metastasis status, and BM, while these variables did not show a statistically significant association with BM in the multivariate analysis. A visual nomogram was thus developed to predict the probability of BM in RCC. The established nomogram had high accuracy and sensitivity in terms of identifying BM in RCC, and its calibration curves also showed good concordance between predicted and observed BM probabilities. Clear cell RCC (ccRCC) patients with a tumor size ≥ 7cm and bone and lung metastasis have higher a risk of BM. Even though in the absence of concerning neurological symptoms, including headache, dizziness, and altered consciousness, targeted brain imaging should be performed for RCC patients with a high risk of BM during a clinical follow-up. Generally, biological characteristics of the tumor play a crucial role in disease progression and are thought to be closely correlated with metastasis formation [25]. In a previous study, tumor size was proven to be a significant factor in the progression of distant metastasis for RCC patients, and there was a linear positive correlation between tumor size and the metastatic rate [26]. This observation was also confirmed in our study. RCC patients with larger tumor diameters (>7 cm) showed a higher risk of BM in multivariate analysis. In a retrospective series focused on patients with metastatic RCC in the brain, Shuch et al. suggested that clear cell RCC appeared to be the predominant histological type that metastasized to the brain, and this type accounted for 92.7% of RCC patients with BM [27]. Suarez-Sarmiento et al. also found that clear cell RCC patients were prone to developing BM [28]. Our study also identified histological type as an independent BM-related risk factor for RCC patients, which was similar to previous observations. Furthermore, in 2011, Verma et al. reported that lung metastasis was associated with a significantly high risk of developing BM in RCC patients [12]. Among 44 RCCBM patients, almost 90% had lung metastasis at diagnosis, and only 2.5% of RCC patients without lung metastasis ultimately developed BM. Therefore, it is worth investigating whether other extracranial metastases promote the development of BM in a synergistic manner. Our study also found that lung and bone metastatic status were the independent risk factors for BM in RCC patients. Although we found that RCC patients with lung or bone metastasis face a higher risk of BM, the complex mechanism remains unclear.

To the best of our knowledge, this is the first report on the construction of a practical nomogram for accurately predicting the probability of BM in RCC. Our comprehensive nomogram could be used as a supportive graphic tool to identify RCC patients with a considerably high propensity for BM, which not only will contribute to the more reasonable allocation of medical resources but will also enable further improvements in the prognosis and quality of life of RCC patients. The established nomogram demonstrated high accuracy and sensitivity for identifying BM in RCC, and its calibration curves also showed good concordance between predicted and observed BM probabilities. Even more notably, the ROC analysis in our study confirmed that the discriminative power of the nomogram was better than that of any the independent risk factors, again illustrating the significance of a comprehensive predictive model (Figure 5). In addition, the identified independent BM-related factors in our study are easily accessible in the daily clinic.

However, there were also some limitations of this study that should be mentioned. First, some selection bias was inevitable due to the retrospective design of our study. Second, we could not evaluate patients who developed BM after being diagnosed with RCC and during the disease course because detailed follow-up data about BM were not recorded in the SEER database. Third, the nomogram provided a relative reference for clinical doctors. In addition to the independent variables included in the nomogram, several other potentially significant details were missing in the current study, such as some laboratory data, molecular biological information of tumors, and clinical symptoms. Fourth, although it highlighted several of the most common metastatic sites of RCC patients, it did not provide data regarding other important types of metastases, such as adrenal metastases. The severity of metastases to other organs could not be obtained from the SEER database.

## 5. Conclusion

The present study identified tumor size, histological type, bone metastatic status, and lung metastatic status as independent risk factors of BM in RCC patients. These independent BM-associated risk factors were integrated to build a diagnostic nomogram to identify RCC patients with a high risk of BM.

## Figures and Tables

**Table 1:** Demographics and clinical characteristics of RCC patients.

Variables	Training cohort (*N* = 29805)		Validation cohort (*N* = 12772)	
	Without BM (*N* = 29652)	With BM (*N* = 153)	Without BM (*N* = 12697)	With BM (*N* = 75)
*Age (years)*				
<45	2943 (9.2%)	11 (7.2%)	1238 (9.8%)	3 (4.0%)
45-65	15279 (51.5%)	92 (60.1%)	6553 (51.6%)	50 (66.7%)
>65	11430 (38.5%)	50 (32.7%)	4906 (38.6%)	22 (29.3%)
*Race*				
Black	3258 (10.9%)	7 (4.6%)	1430 (11.3%)	1 (1.3%)
Other^a^	1929 (6.5%)	14 (9.2%)	835 (6.6%)	8 (10.7%)
White	24465 (82.5%)	132 (86.2%)	10432 (82.1%)	66 (88.0%)
*Sex*				
Female	10721 (36.2%)	45 (29.4%)	4556 (35.9%)	21 (28.0%)
Male	18931 (63.8%)	108 (70.6%)	8141 (64.1%)	54 (72.0%)
*Histological type*				
pRCC	4637 (15.6%)	3 (2.0%)	2025 (15.9%)	2 (2.7%)
cRCC	1548 (5.2%)	2 (1.3%)	652 (5.1%)	0 (0.0%)
ccRCC	23403 (78.9%)	147 (96.0%)	9989 (78.7%)	72 (96.0%)
cdRCC	64 (0.2%)	1 (0.7%)	31 (0.2%)	1 (1.3%)
*Histological grade*				
Grade I	3416 (11.5%)	6 (4.0%)	1392 (11.0%)	1 (1.3%)
Grade II	15651 (52.8%)	41 (26.8%)	6931 (54.6%)	21 (28.0%)
Grade III	8757 (29.5%)	64 (41.8%)	3622 (28.5%)	37 (49.4%)
Grade IV	1828 (6.2%)	42 (27.4%)	752 (5.9%)	16 (21.3%)
*Laterality*				
Left	14638 (49.4%)	79 (51.6%)	6205 (48.9%)	40 (53.3%)
Right	15014 (50.6%)	74 (48.4%)	6492 (51.1%)	35 (46.7%)
*T stage*				
T1-2	23661 (79.8%)	65 (42.5%)	10144 (79.9%)	31 (41.3%)
T3-4	5591 (18.9%)	88 (57.5%)	2553 (20.1%)	44 (58.7%)
*N stage*				
N0	28843 (97.3%)	119 (77.8%)	12348 (97.3%)	61 (81.3%)
N1-2	809 (2.7%)	34 (22.2%)	349 (2.7%)	14 (18.7%)
*Bone metastasis*				
No	29171 (98.4%)	111 (72.5%)	12505 (98.5%)	49 (65.3%)
Yes	481 (1.6%)	42 (27.5%)	192 (1.5%)	26 (34.7%)
*Liver metastasis*				
No	29472 (99.4%)	138 (90.2%)	12620 (99.4%)	69 (92.0%)
Yes	180 (0.6%)	15 (9.8%)	77 (0.6%)	6 (8.0%)
*Lung metastasis*				
No	28719 (96.9%)	55 (35.9%)	12300 (96.9%)	23 (30.7%)
Yes	933 (3.1%)	98 (64.1%)	397 (3.1%)	52 (69.3%)
*Tumor size*				
≤4 cm	13608 (45.9%)	5 (3.3%)	6289 (49.5%)	8 (10.7%)
4-7 cm	9269 (31.3%)	23 (15.0%)	3778 (29.8%)	17 (22.7%)
7-10 cm	4069 (13.7%)	60 (39.2%)	1603 (12.6%)	25 (33.3%)
>10	2706 (9.1%)	65 (42.5%)	1027 (8.1%)	25 (33.3%)
*Insurance status*				
Insured^b^	28868 (97.4%)	148 (96.7%)	12390 (97.6%)	72 (96.0%)
Uninsured	784 (2.6%)	5 (3.3%)	307 (2.4%)	3 (4.0%)
*Marital status*				
Married	19206 (64.8%)	92 (60.1%)	8335 (65.6%)	58 (77.3%)
Unmarried^c^	10446 (35.2%)	61 (39.9%)	4362 (34.4%)	17 (22.7%)

^a^American Indian, native Alaskan and Asian, and Pacific Islander. ^b^Any medicaid, insured, and insured/not specific. ^c^Unmarried, separated, single, widow, and divorced. pRCC: papillary renal cell carcinoma; cRCC: chromophobe renal cell carcinoma; ccRCC: clear cell renal cell carcinoma; cdRCC: collecting duct renal cell carcinoma.

**Table 2:** Logistic regression analysis of independent risk factors of BM in RCC patients.

Variable	Univariate analysis		Multivariate analysis	
	OR (95% CI)	*P* value	OR (95% CI)	*P* value
*Age (years)*				
<45	Reference			
45-65	1.611 (0.861-3.014)	0.136		
>65	1.170 (0.609-2.251)	0.637		
*Race*				
Black	Reference			
Other^a^	3.378 (1.361-8.384)	0.009		
White	2.511 (1.173-5.375)	0.018		
*Sex*				
Female	Reference			
Male	1.359 (0.959-1.926)	0.084		
*Histological type*				
pRCC	Reference			
cRCC	1.997 (0.333-11.962)	0.449	1.958 (0.321-11.938)	0.467
ccRCC	9.709 (3.094-30.464)	<0.001	5.239 (1.650-16.638)	0.005
cdRCC	24.151 (2.479-235.310)	0.006	3.523 (0.336-36.896)	0.293
*Histological grade*				
Grade I	Reference			
Grade II	1.491 (0.633-3.516)	0.361		
Grade III	4.161 (1.800-9.617)	0.001		
Grade IV	13.081 (5.550-30.829)	<0.001		
*Laterality*				
Left	Reference			
Right	0.913 (0.665-1.255)	0.576		
*T stage*				
T1-2	Reference			
T3-4	5.347 (3.876-7.377)	<0.001		
*N stage*				
N0	Reference			
N1-2	10.186 (6.914-15.007)	<0.001		
*Bone metastasis*				
No	Reference			
Yes	22.947 (15.909-33.100)	<0.001	2.924 (1.937-4.416)	<0.001
*Liver metastasis*				
No	Reference			
Yes	17.797 (10.241-30.928)	<0.001		
*Lung metastasis*				
No	Reference			
Yes	54.847 (39.172-76.795)	<0.001	15.649 (10.529-23.259)	<0.001
*Tumor size*				
≤4 cm	Reference			
4-7 cm	6.753 (2.567-17.770)	<0.001	4.971 (1.880-13.146)	0.001
7-10 cm	40.132 (16.106-99.998)	<0.001	14.997 (5.865-38.346)	<0.001
>10	65.375 (26.301-162.501)	<0.001	14.620 (5.631-37.962)	<0.001
*Insurance status*				
Insured^b^	Reference			
Uninsured	1.244 (0.509-3.041)	0.632		
*Marital status*				
Married	Reference			
Unmarried^c^	1.219 (0.881-1.686)	0.32		

^a^American Indian, native Alaskan and Asian, and Pacific Islander. ^b^Any medicaid, insured, and insured/not specific. ^c^Unmarried, separated, single, widow, and divorced. pRCC: papillary renal cell carcinoma; cRCC: chromophobe renal cell carcinoma; ccRCC: clear cell renal cell carcinoma; cdRCC: collecting duct renal cell carcinoma.

## Data Availability

The datasets generated during and/or analyzed during the current study are available from the corresponding author on reasonable request.

## Abbreviations

RCC: Renal cell carcinoma
BM: Brain metastasis
mRCC: Metastatic renal cell carcinoma
SEER: Surveillance, Epidemiology, and End Results
ROC: Receiver operating characteristic
DCA: Decision curve analysis
AUC: Area under the curve.
